# Prevalence of Polypharmacy and Potentially Inappropriate Medications Use in Elderly Chinese Patients: A Systematic Review and Meta-Analysis

**DOI:** 10.3389/fphar.2022.862561

**Published:** 2022-06-20

**Authors:** Fangyuan Tian, Zhaoyan Chen, Jinhui Wu

**Affiliations:** ^1^ Department of Pharmacy, National Clinical Research Center for Geriatrics, West China Hospital, Sichuan University, Chengdu, China; ^2^ Department of Epidemiology and Health Statistics, West China School of Public Health and West China Fourth Hospital, Sichuan University, Chengdu, China; ^3^ Department of Geriatrics. National Clinical Research Center for Geriatrics, West China Hospital, Sichuan University, Chengdu, China

**Keywords:** polypharmacy, potentially inappropriate medication, elderly, Chinese patients, meta-analysis

## Abstract

**Objectives:** Polypharmacy and potentially inappropriate medication (PIM) use among elderly Chinese patients have not yet been investigated by systematic review and meta-analysis. The purposes of this study were to investigate the prevalence of polypharmacy and PIM use and the risk factors associated with PIM use in elderly Chinese patients.

**Methods:** Databases including PubMed, EMBase, and Web of Science were searched to collect studies which used Chinese criteria, Beers criteria, or STOPP criteria to evaluate the PIM status of elderly Chinese patients from inception to August 2021 (PROSPERO Code No: CRD42021262821). Observational studies reporting the prevalence of polypharmacy and PIM use in elderly Chinese patients were meta-analyzed. The pooled prevalence and risk ratio (RR) were calculated with a 95% confidence interval (CI).

**Results:** A total of 8 articles involving 4,558,786 patients were included. The overall prevalence of polypharmacy (concomitant use of more than 5 medicines) and PIM use pooled by meta-analysis in Chinese older patients was 48% (95% CI: 0.17, 0.79, *p* = 0.003) (inpatients 73%, outpatients 23%) and 39% (95% CI: 0.25, 0.54, *p* < 0.001) (inpatients 50%, outpatients 29%), respectively. Polypharmacy (RR: 2.03, 95% CI: 1.13, 3.64) was significantly associated with PIM use.

**Conclusion:** This meta-analysis demonstrated a high prevalence of polypharmacy and PIM use among elderly patients in China. Affected by the quantity and quality of the included studies, the aforementioned conclusions need to be confirmed by large samples and high-quality studies.

## 1 Introduction

According to statistics from the World Health Organization (WHO), the number of older adults has been constantly rising worldwide, and China is currently the country with the largest elderly population in the world. As elderly adults grow older, their physical functions deteriorate, and they are often prone to multiple diseases and need to take more drugs. (Cojutti et al., 2016). Polypharmacy (defined as more than five drugs) has become common among the elderly because they suffer from a variety of diseases, especially chronic diseases. The increase in the number of drugs not only brings therapeutic effects to the elderly but also brings risks due to the interaction between drugs (Scott et al., 2015). These negative effects may reduce the quality of life of the elderly, increase the risk of falls, prolong the length of hospital stay, and further increase the risk of drug-induced diseases (Hamilton et al., 2011; Cahir et al., 2014; Davies and O’Mahony, 2015).

Among the drugs used by the elderly, some are relatively safe, while others are used on the elderly for some reason, resulting in a high risk of adverse outcomes. These drugs are called potentially inappropriate medications (PIMs) (Tian et al., 2021a). The Beers criteria were the first expert consensus on geriatric PIMs (Beers et al., 1991). The American Geriatrics Society has undertaken their sixth iteration (American Geriatrics Society Beers Criteria® Update Expert Panel, 2019). University College Cork organized experts from many disciplines to formulate the screening tool of old peoples prescriptions/screening tool to alert to the right treatment (STOPP/START criteria) through the Delphi method, and the second edition was updated in 2014 (O'Mahony et al., 2015; O'Mahony, 2020). These two criteria have been widely used to evaluate the PIMs’ use in the elderly population around the world. China has formulated the criteria for judging the potentially inappropriate medication use by older adults in 2016.

There are also some studies that have applied these criteria to investigate the prevalence of polypharmacy and PIM use in Chinese elderly patients. Until now, some systematic reviews and meta-analyses have been conducted on polypharmacy or PIM use in the elderly (Bhagavathula et al., 2022; Davies et al., 2020; Mohamed et al., 2020; Xing et al., 2019; Liew et al., 2019). Due to the focus of each study being different, this limited the applicability and interpretability of existing results in China. To overcome these limitations, we conducted the study on Chinese elderly patients to provide relevant evidence.

## 2 Methods and Materials

### 2.1 Search Strategy

This study was performed according to the Preferred Reporting Items for Systematic Reviews and Meta-analysis guidelines (Moher et al., 2015). This systematic review and meta-analysis was registered on PROSPERO (CRD42021262821). We searched PubMed, EMBase, and the Web of science from inception to August 25, 2021. For PubMed, the search items included: [“Polypharmacy” (MeSH Terms)] OR [“Potentially Inappropriate Medication” (Title/Abstract)] OR [“Potentially Inappropriate Prescription” (Title/Abstract)] OR [“Inappropriate Medication” (Title/Abstract)] OR [“Inappropriate Prescription” (Title/Abstract)] OR [“Inappropriate Prescribing” (Title/Abstract)] OR [“Inappropriate Drug Use” (Title/Abstract)] AND [“Chinese” (Title/Abstract) OR “China” (Title/Abstract)]. For EMBase, the search items included: (“Polypharmacy”) [Title/Abstract] OR (“exp Polypharmacy”) OR [“Potentially Inappropriate Medication” (Title/Abstract)] OR (“exp Potentially Inappropriate Medication”) AND [“Chinese” (Title/Abstract) OR “exp Chinese”]. {[TI=(Polypharmacy)] OR TI=(Potentially Inappropriate Medication)} AND TI=(Chinese). Observational studies conducted on Chinese elderly patients were published in English and reported both the prevalence of polypharmacy and the prevalence of PIM use. The search string used medical subject heading and nonmedical subject heading terms.

### 2.2 Selection Criteria and Data Extraction

The studies met the following criteria: 1) reported both prevalence of polypharmacy and the prevalence of PIM use in Chinese elderly patients; 2) risk factors that increase PIM use in Chinese elderly patients. Studies were excluded if they 1) did not report the prevalence of polypharmacy or PIM use in Chinese elderly patients; 2) duplicate studies, reviews, case reports, interventional studies, and meta-analyses were also excluded.

### 2.3 Selection of Studies

Two reviewers (FY Tian and ZY Chen) screened the titles and abstracts of the literature back-to-back. The senior investigator (JH Wu) reviewed the first 50 references independently. The level of agreement was 90% with only five discrepancies, which were discussed between the three reviewers to arrive at a consensus. The remaining studies were then further reviewed by the two reviewers.

### 2.4 Quality Assessment

The Agency for Healthcare Research and Quality (AHRQ) was used to evaluate the quality of the cross-sectional study (Hu et al., 2015). The AHRQ assesses the representativeness of the source of information, inclusion and exclusion criteria, indicates time period, indicates whether or not subjects were consecutive, indicates if evaluators of subjective components of study were masked to other aspects of the status of the participants, describes any assessments undertaken for quality assurance purposes, explains any patient exclusions from analysis, describes how confounding was assessed and/or controlled, explains how missing data were handled in the analysis, summarizes patient response rates and completeness of data collection, clarifies follow-up (Chen et al., 2018). The AHRQ scores range from 0 (lowest grade) to 11 (highest grade). Studies scoring eight or above were considered high quality, and those with scores below four were of low quality.

### 2.5 Statistical Analysis

A meta-analysis (proportions) was performed using STATA software. Pooled prevalence was reported as percentages with 95% CI, considering the variations in the true effect size across the population. Der Simonian and Laird’s random-effects model was applied. A pooled relative ratio was calculated for each study to determine the association between various patient factors and the risk of use of PIMs (Bhagavathula et al., 2022). Statistical heterogeneity was assessed by determining the I^2^ statistics. When I^2^ is > 50% (indicates high heterogeneity), subgroup analysis and sensitivity analysis were performed to investigate the source of heterogeneity.

## 3 Results

### 3.1 Basic Information of Studies

**FIGURE 1:**
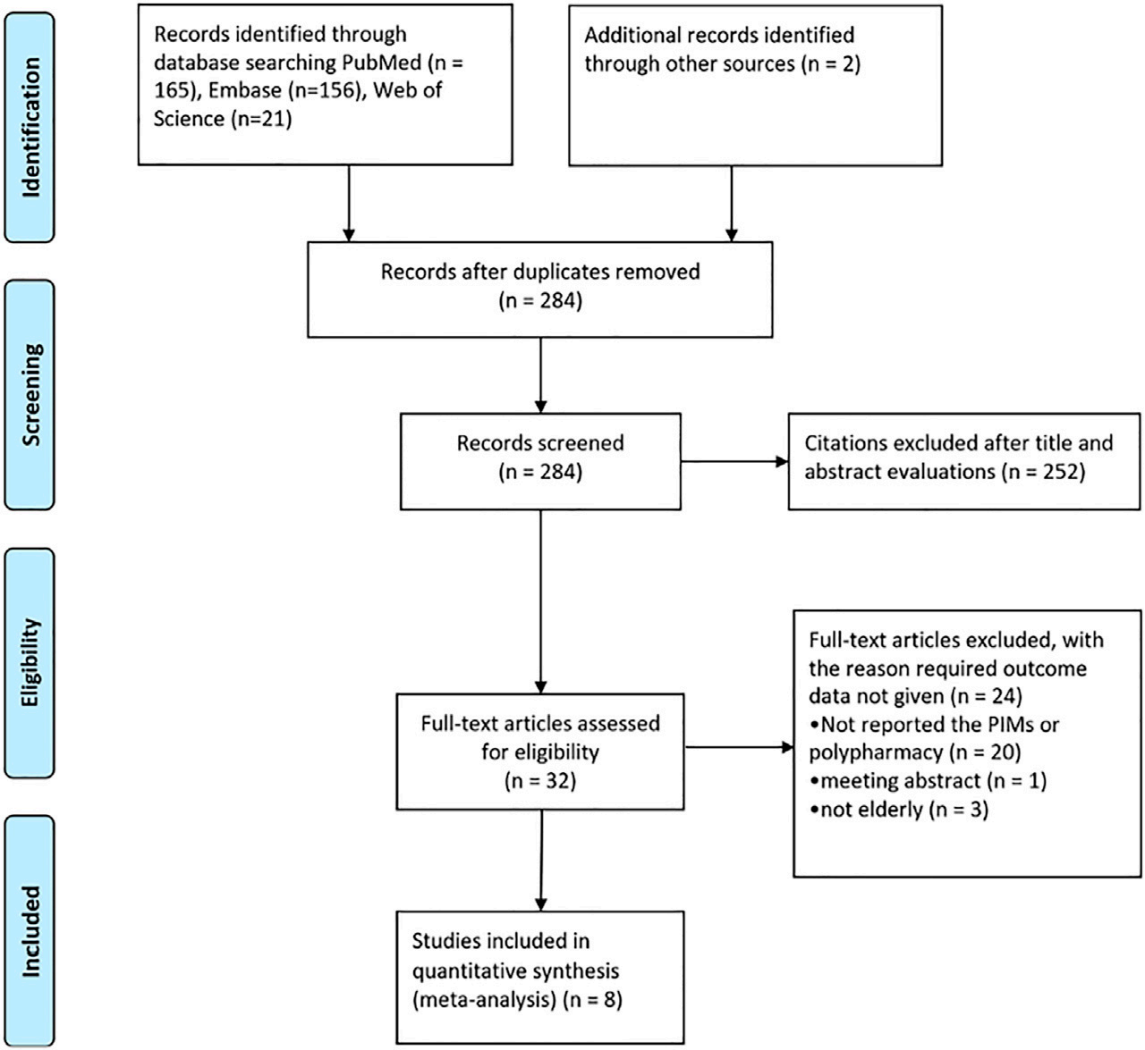
Diagram of the literature selection.

A total of 344 records were identified through PubMed, EMBase, and Web of Science. 60 duplicates were removed using Endnote. After the first round of screening, 252 articles were excluded from 284 literatures, and the remaining 32 articles were put through a second round of screening. 24 articles were excluded as they did not report the PIMs or polypharmacy, meeting abstracts, and the population was not elderly. Finally, 8 studies (Fu et al., 2020; Huang et al., 2020; Ma et al., 2020; Zheng et al., 2020; Tian et al., 2021a; He et al., 2021; Li et al., 2021; Zhao et al., 2021) were included in the study (Figure 1).

The included studies, comprising a total of 4558786 participants, are representative of the Chinese elderly population. Four studies were on outpatients, and four studies were on inpatients. The majority of studies were conducted in the Chinese provincial capital city and used a version of the Beers criteria, STOPP criteria, and Chinese criteria. According to the AHRQ, the average score of studies was 7, which indicates moderate quality (Table 1).

**TABLE 1:** Characteristics of included studies.

Article	Location	Study design	Mean age (y)	Sample size	Setting	Male (%)	PIM criteria applied	Prevalence (%)		Quality of studies
								Polypharmacy (≥5 drugs used)	PIM use	
Zhao et al., (2021)	Beijing	Cross-sectional	71.5 ± 7.2	447	Inpatients	60	2019 Beers criteria	65.5	38	7
Tian et al., (2021a)	Chengdu	Cross-sectional	78	12,005	Outpatients	59.61	2019 Beers criteria, 2015 Beers criteria	24.15	2019 Beers criteria: 34.39, 2015 Beers criteria: 30.98	7
He et al., (2021)	Nanjing	Cross-sectional	73.74 ± 6.98	6,424	Inpatients	58.92	2019 Beers criteria, 2015 Beers criteria	94.05	2019 Beers criteria: 64.80, 2015 Beers criteria: 64.31	8
Li et al., (2021)	Suzhou	Cross-sectional	74.64 ± 7.32	8,235	Outpatients	51.86	2019 Beers criteria, 2017 Chinese criteria	19.09	2019 Beers criteria: 32.16, 2017 Chinese criteria: 37.07	6
Zheng et al., (2020)	Xi’an	Cross-sectional	——	315	Inpatients	46.3	2019 Beers criteria	96.5	49.2	7
Ma et al., (2020)	Beijing	Cross-sectional	73	662	Inpatients	54.53	STOPP/START V2, STOPP/START V1	90.4	STOPP/START V2: 47.7, STOPP/START V1: 36.1	7
Huang et al., (2020)	Changsha	Cross-sectional	86	1,874	Outpatients	80	2019 Beers criteria, 2017 Chinese criteria	44.7	2019 Beers criteria: 35, 2017 Chinese criteria: 50.6	7
Fu et al., (2020)	Beijing	Cross-sectional	72.0 ± 8.9	4,528,824	Outpatients	44.1	2017 Chinese criteria	5.5	14.1	7

### 3.2 Prevalence of Polypharmacy and Potentially Inappropriate Medication use in the Chinese Elderly Patients

**FIGURE 2:**
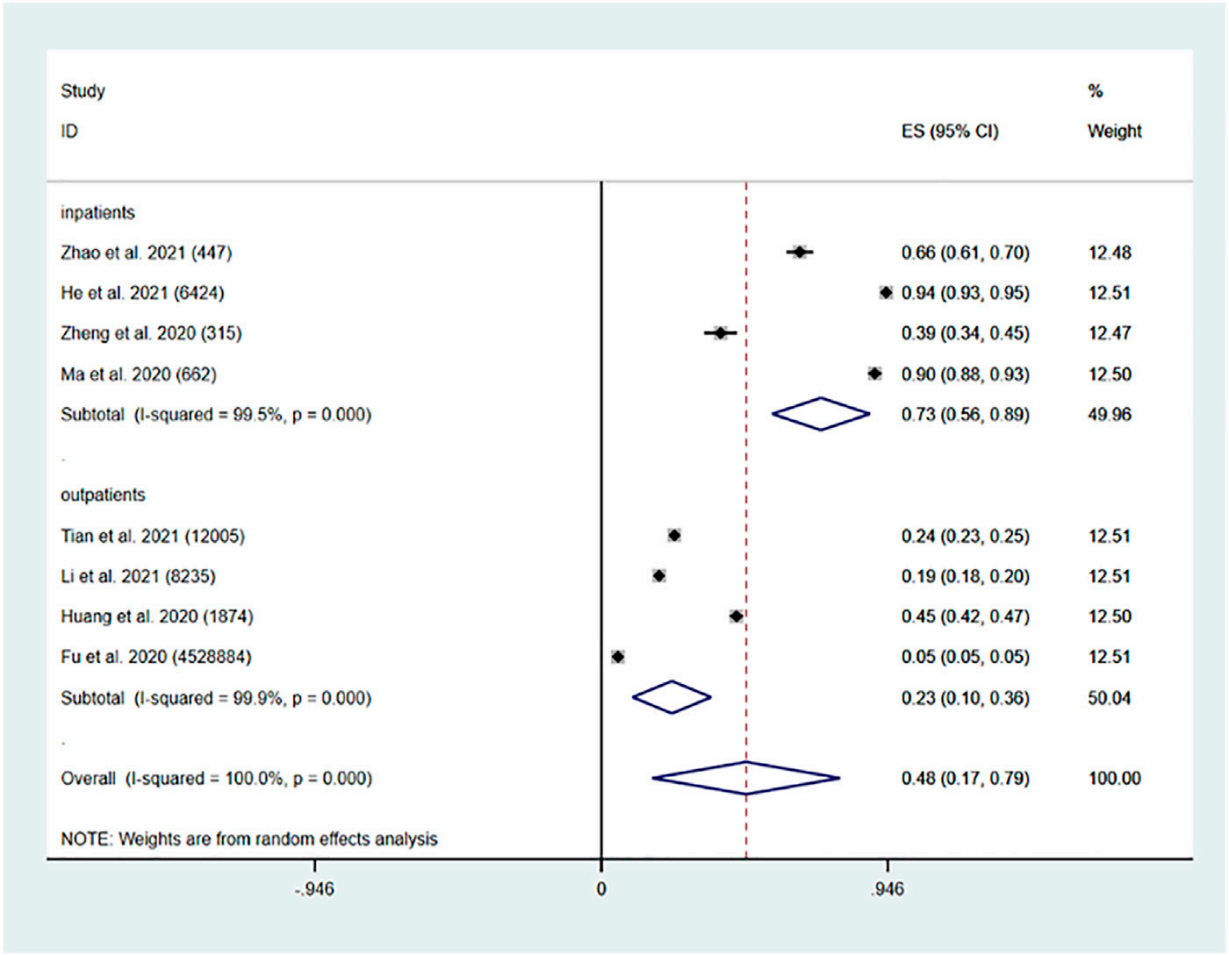
Prevalence of polypharmacy in the Chinese older population.

Eight studies (Fu et al., 2020; Huang et al., 2020; Ma et al., 2020; Zheng et al., 2020; Tian et al., 2021b; He et al., 2021; Li et al., 2021; Zhao et al., 2021) showed the prevalence of polypharmacy in the elderly Chinese population. Four studies were on outpatients (Fu et al., 2020; Huang et al., 2020; Tian et al., 2021b; Li et al., 2021); and four studies were on inpatients (Ma et al., 2020; Zheng et al., 2020; He et al., 2021; Zhao et al., 2021). The pooled prevalence of polypharmacy in China was found to be 48% (95% CI: 0.17, 0.79, *p* = 0.003). The pooled prevalence of polypharmacy in Chinese older inpatients was 73% (95% CI: 0.56, 0.89, *p* < 0.001). The pooled prevalence of polypharmacy in Chinese older outpatients was 23% (95% CI: 0.10, 0.36, *p* < 0.001) (Figure 2).

**FIGURE 3:**
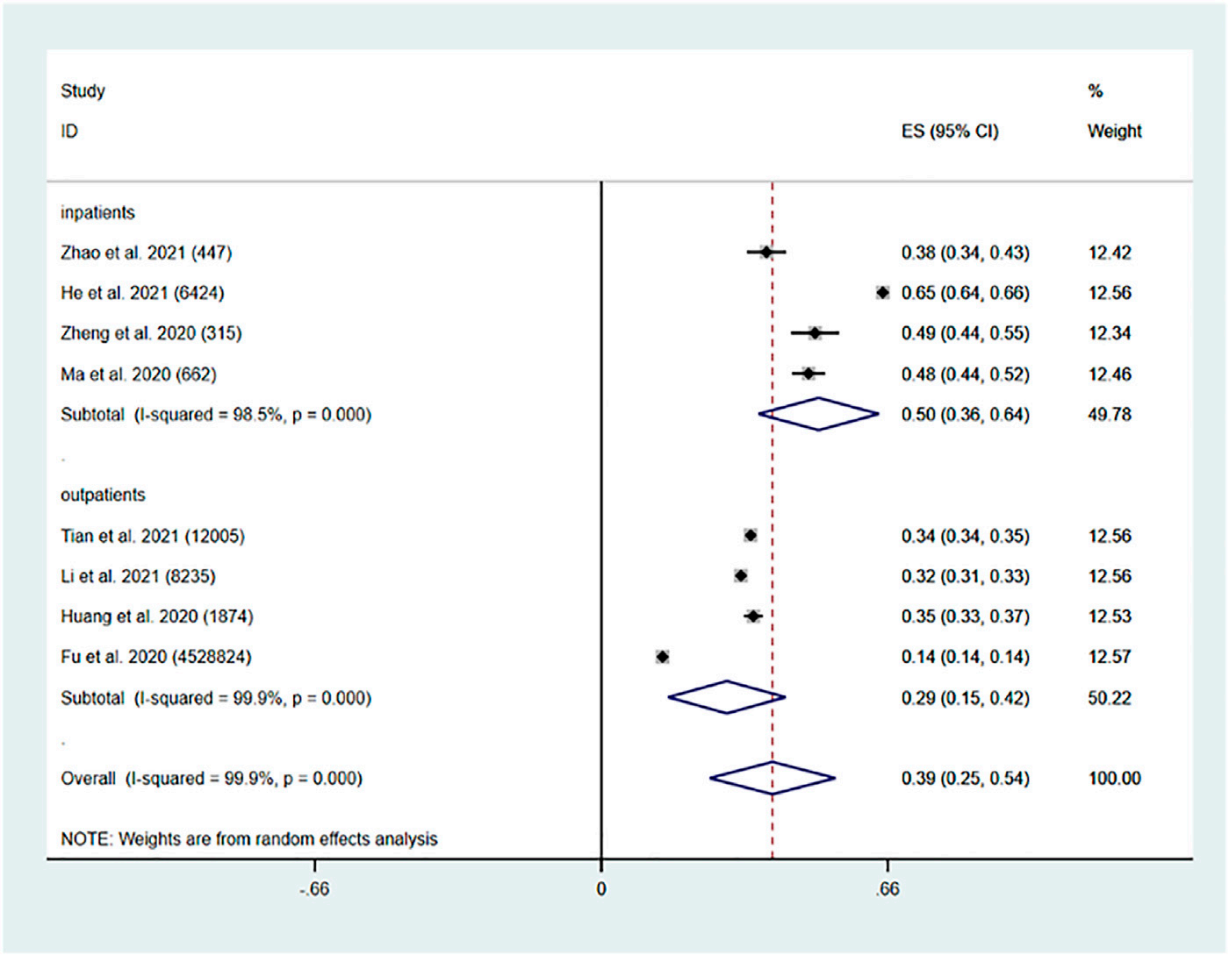
Prevalence of PIM use in the Chinese elderly population.

The pooled prevalence of PIM use in China was found to be 39% (95% CI: 0.25, 0.54, *p* < 0.001). The pooled prevalence of polypharmacy in Chinese older inpatients was 50% (95% CI: 0.36, 0.64, *p* < 0.001). The pooled prevalence of PIM use in Chinese older outpatients was 29% (95% CI: 0.15, 0.42, *p* < 0.001) (Figure 3).

### 3.3 Risk Factors Associated With Potentially Inappropriate Medications Use

#### 3.3.1 Age

Four studies (Tian et al., 2021a; He et al., 2021; Li et al., 2021; Zheng et al., 2020) reported the association of older age and the risk of PIM use. With increasing age, the risk of PIM use is also higher, but no statistically significant increase in the risk of PIM use was confirmed for higher age categories. The stratified meta-analysis showed an increased risk (75∼84 years old vs. 65∼74 years old) of 7% PIM use exposure (RR: 1.07, 95% CI: 0.99, 1.16, *p* = 0.107), (≥85 years old vs. 75∼84 years old) of 11% PIM use exposure (RR: 1.11, 95% CI: 0.97, 1.26, *p* = 0.120), (≥85 years old vs. 65∼74 years old) of 18% PIM use exposure (RR: 1.18, 95% CI: 0.98, 1.41, *p* = 0.082) (Table 2).

**TABLE 2:** Factors associated with increased risk of PIM use.

	Characteristics	Number of studies	Relative ratio, RR	95% CI	*P*
Age	75∼84 vs. 65∼74	4	1.07	0.99, 1.16	0.107
	≥85 vs. 75∼84	4	1.11	0.97,1.26	0.120
	≥85 vs. 65∼74	4	1.18	0.98, 1.41	0.082
Gender	7	0.95	0.89, 1.01	0.08	
Different criteria	2019 Beers criteria vs. 2015 Beers criteria	2	1.04	0.97, 1.12	0.267
	2017 Chinese criteria vs. 2019 Beers criteria	2	1.20	1.03, 1.39	0.020
Polypharmacy	7	2.03	1.13, 3.64	0.018	

#### 3.3.2 Gender

Seven studies (Fu et al., 2020; Huang et al., 2020; Ma et al., 2020; Zheng et al., 2020; Tian et al., 2021a; He et al., 2021; Li et al., 2021) reported the association between sex differences and risk of PIM use. The meta-analysis revealed that PIM use in the older population was not associated with gender differences (RR: 0.95, 95% CI: 0.89, 1.01, *p* = 0.080) (Table 2).

#### 3.3.3 Different Criteria

Five studies (Huang et al., 2020; Ma et al., 2020; Tian et al., 2021b; He et al., 2021; Li et al., 2021) reported the association of different criteria with risk of PIM use, two studies (Tian et al., 2021b; He et al., 2021) reported an association between 2019 Beers criteria and 2015 Beers criteria, two studies (Huang et al., 2020; Li et al., 2021) reported an association between 2019 Beers criteria and 2017 Chinese criteria, and one study (Ma et al., 2020) reported an association between STOPP/START V2 criteria and STOPP/START V1 criteria. The meta-analysis revealed that 2019 Beers criteria are more sensitive than 2015 Beers criteria (RR: 1.04, 95% CI: 0.97, 1.12, *p* = 0.267) with no significant difference. 2017 Chinese criteria is more sensitive than 2019 Beers criteria with a significant difference (RR: 1.20, 95% CI: 1.03, 1.39, *p* = 0.02). STOPP/START V2 criteria are more sensitive than STOPP/START V1 criteria (47.7 vs. 36.1%) (Table 2).

#### 3.3.4 Association of Polypharmacy With the Risk of Potentially Inappropriate Medication Use

Seven studies (Fu et al., 2020; Huang et al., 2020; Ma et al., 2020; Zheng et al., 2020; Tian et al., 2021b; He et al., 2021; Li et al., 2021) investigated the association of polypharmacy with the risk of PIM exposure in the Chinese older population. The pooled effect estimate indicated a significant risk of PIM use (RR: 2.03, 95% CI: 1.13, 3.64, *p* = 0.018) in polypharmacy patients (Table 2).

## 4 Discussion

Our study is the first systematic literature review and meta-analysis to describe the pooled prevalence of polypharmacy, PIM use, and risks of PIM use in China. Based on our evaluation of eight studies, the overall prevalence of polypharmacy in the older population was 48%. These elderly patients came from six cities in China, and the prevalence of polypharmacy ranged from 5.5 to 96.5%. The pooled prevalence of polypharmacy of elderly Chinese inpatients was 73% and that of elderly Chinese outpatients was 23%. Five studies reported the prevalence of PIM under two different criteria, while three studies reported the prevalence of PIM under one criterion. The 2019 Beers criteria were the most widely used criteria. A study on the use of prescription drugs by adults in the United States reported that between 1999 and 2012, the polypharmacy of patients aged 65 and over accounted for 24–39% (Kantor et al., 2015). Another study from Europe found that 32.1% of the elderly took five or more drugs every day (Midão et al., 2018). In Australia, a study about the prevalence of polypharmacy in the elderly population was 43–91% (Page et al., 2019). In Asia, the studies demonstrated that prevalence of polypharmacy of elderly patients could be 86.4% in South Korea (Kim et al., 2014) and 20% in Japan (Amano et al., 2020). The overall prevalence of PIM use was 39% in our study. According to a recent meta-analysis, the pooled prevalence of PIM use in elderly patients in developing countries was 37% (Bhagavathula et al., 2022) and that in developed countries was 33.3% (Liew et al., 2020). Our meta-analysis revealed a higher pooled prevalence of PIM use in China (39%) than in other developing countries or developed countries. As China becomes an aging society, many elderly people suffer from a variety of chronic diseases and take multiple drugs, which may be potentially inappropriate. Furthermore, polypharmacy is more frequently observed in inpatients (73%) than outpatients (23%). This is different from another study about the prevalence of polypharmacy observed in two categories (Bhagavathula et al., 2021).

From the results obtained in the study, the population aging and rising trends of the risk of PIM use was demonstrated for higher age categories. Our study revealed that non-significant PIM use in Chinese elderly patients is associated with gender differences, but female patients look like they have a higher risk of PIMs than male patients, consistent with research at home and abroad (Al-Azayzih et al., 2019; Nam et al., 2016). Chinese criteria are more sensitive than other criteria, which may be because Chinese criteria were made based on the drug utilization of the Chinese elderly population, so they may be more suitable for the Chinese population. Significant findings between the risk of polypharmacy and PIM use in the study may lead to adverse clinical outcomes. The findings of current studies revealed that polypharmacy was associated with negative outcomes. In general, polypharmacy may lead to negative outcomes, including falls, frailty, and low quality of life (Gutiérrez-Valencia et al., 2018; Leelakanok et al., 2017; Fried et al., 2014; Maher et al., 2014). One systematic review even showed that polypharmacy was associated with death (Leelakanok et al., 2017).

PIM use led to high morbidity and mortality in the elderly, and the prevalence of polypharmacy increased as well (Achterhof et al., 2020). Deprescribing is an established management strategy to minimize polypharmacy and PIM use (Wu et al., 2021). The understanding of the clinical efficacy of reducing polypharmacy in the elderly is limited, especially in Chinese. The benefits and sustainability of deprescribing intervention in unplanned hospital admission or death of elderly patients are still unclear (Rieckert et al., 2020). One study showed that deprescribing or not has little effect on reducing unnecessary medication (Ibrahim et al., 2021). Perhaps sometimes, polypharmacy is appropriate, and sometimes, polypharmacy is inappropriate. Reducing PIM use may be more meaningful. Some studies suggested that pharmacist-led deprescribing intervention in the elderly was effective in reducing unnecessary medications (Verrue et al., 2009; Tjia et al., 2013; Stuhec and Lah, 2021). Clinical pharmacists represent a powerful approach to unnecessary polypharmacy and PIM reduction in elderly patients in Europe (Stuhec et al., 2019; Stuhec et al., 2021).

The present study suggests that interventions targeting unnecessary polypharmacy and PIM use may improve health outcomes among the elderly (Mekonnen et al., 2021). Deprescribing is not difficult for most elderly people to accept, and it can improve their medication compliance. However, for the elderly over 80 years old, the effect of deprescribing is relatively poor and could not reduce mortality, which may be related to the poor physical condition of the patients (Page et al., 2016). The comprehensive geriatric assessment (CGA) on reducing unnecessary polypharmacy and PIM use demonstrated that this could be decreased by CGA in elderly patients. Furthermore, this will have beneficial effects on economic parameters due to decreasing drug-related healthcare costs (Unutmaz et al., 2018). However, no relevant research has been reported in China. So, it is necessary to establish standardized tools to reduce unnecessary polypharmacy and PIM use in Chinese older patients, especially in inpatients. The impact of intervention measures on the health outcomes of Chinese elderly patients still needs to be verified.

## 5 Limitations

This study was to integrate the evidence of polypharmacy and PIM use in Chinese elderly patients. However, there were some limitations. First, factors such as disease distribution, doctors’ diagnostic level, and prescribing habits vary widely across the hospitals in China, which may influence generalization of results. Second, the majority of studies were conducted in the Chinese provincial capital city, so results of other urban and rural areas are unclear. Third, few studies were included in this study and the overall quality was general, but most of them were short-term studies. Therefore, the aforementioned conclusions need to be confirmed by large-scale and high-quality studies.

## 6 Conclusion

This study revealed a high prevalence of polypharmacy and PIM use in Chinese elderly patients, which was 48% (inpatients 73%, outpatients 23%) and 39% (inpatients 50%, outpatients 29%). PIMs’ use in the elderly was dependent on polypharmacy, and Chinese criteria were more sensitive than Beers’ criteria for Chinese elderly patients.

## Data Availability

The original contributions presented in the study are included in the article/supplementary material; further inquiries can be directed to the corresponding author.
